# Long-Term Variation Characteristics and Health Risks of Atmospheric Hg in the Largest City in Northwestern China

**DOI:** 10.3390/toxics12120935

**Published:** 2024-12-23

**Authors:** Yuqi Pang, Hongmei Xu, Mengyun Yang, Bin Zhang, Liyan Liu, Sulin Chen, Jing Xue, Hui Zhang, Zhenxing Shen

**Affiliations:** 1 Department of Environmental Science and Engineering, Xi’an Jiaotong University, Xi’an 710049, China; pangyuqi@stu.xjtu.edu.cn (Y.P.); ymy9966221@stu.xjtu.edu.cn (M.Y.); zhangbin777@stu.xjtu.edu.cn (B.Z.); lly_lly0110@stu.xjtu.edu.cn (L.L.); 667678csl@stu.xjtu.edu.cn (S.C.); zxshen@mail.xjtu.edu.cn (Z.S.); 2 Key Laboratory for Space Bioscience and Biotechnology, School of Life Sciences, Northwestern Polytechnical University, Xi’an 710072, China; xuejing0089@nwpu.edu.cn; 3 State Key Laboratory of Environmental Geochemistry, Institute of Geochemistry, Chinese Academy of Sciences, Guiyang 550081, China; zhanghui1@mail.gyig.ac.cn

**Keywords:** gaseous element mercury (GEM), gaseous oxidized mercury (GOM), seasonal variation, diurnal variation, health risk

## Abstract

In this study, gaseous element mercury (GEM) and gaseous oxidized mercury (GOM) in the atmosphere were continuously observed at a minute resolution from 1 April 2019 to 31 December 2020 in urban Xi’an, the largest central city in Northwestern China. The concentrations of GEM and GOM drastically fluctuated within the ranges of 0.022–297 ng/m^3^ and 0.092–381 pg/m^3^, showing average values of 5.78 ± 7.36 ng/m^3^ and 14.2 ± 20.8 pg/m^3^, respectively. GEM and GOM showed a decreasing trend of 0.121 ng/m^3^ and 0.472 pg/m^3^ per month, respectively, which we believe was mainly caused by anthropogenic sources, especially by a reduction in coal-fired emissions, rather than meteorological factors. The significant positive correlation between GEM and PM_2.5_, SO_2_, NO_2_, and CO, as well as Cr, As, and Pb in PM_2.5_ also proves that. GEM showed a higher concentration at nighttime than daytime, while an M-shaped diurnal trend was observed for GOM. The hazard quotient of GEM for both males and females decreased at a rate of 0.003 per month, and children aged 2–5 were more sensitive to non-carcinogenic health risks. The changing trends, controlling factors, and human health risks of Hg in the atmosphere are necessary and crucial to study for improving our understanding of the impacts of Hg in Northwestern China.

## 1. Introduction

Mercury (Hg) has the characteristics of strong pollution persistence, strong concealment, and easy migration [1]. Hg in the atmosphere exists in three main forms: gaseous element mercury (GEM), gaseous oxidized mercury (GOM), and particle-bound mercury (PBM) [2]. Studies have shown that GEM is the predominant form of Hg, accounting for about 95% of the total Hg [3]. GEM can be transported over long distances, with a long residence time in the atmosphere (about 0.5–2 years), and it can undergo chemical transformation through photochemical oxidation reactions during transport. GEM can react with oxidants such as O_3_ and OH and can be oxidized into GOM [3]. GOM has a high surface reactivity and water solubility, a fast rate of wet and dry sedimentation, and a short residence time in the atmosphere, ranging from a few hours to a few weeks. At the same time, GOM may also be converted to GEM by a reduction reaction with SO_2_ (g) or SO_3_^2−^ (aq) [4].

Natural sources of atmospheric Hg include volcanic emissions, volatile emissions from the ocean, and evaporation from soil and water surfaces [5]. Hg has a large number of anthropogenic sources, such as fossil-fuel-fired power plants, ferrous and non-ferrous metal manufacturing facilities, caustic soda production plants, ore processing facilities, incinerators, cement plants, and chemicals production facilities [6]. Coal burning is the most important anthropogenic source, accounting for about two-thirds of global anthropogenic Hg emissions [7]. Northwestern China is still largely dependent on coal for energy consumption, but in recent years, with the promotion of new energy sources, the continuous innovation of tail gas purification technology in coal-fired industries, and the tightening of emission standards, the Hg concentration in the region has been significantly reduced [8,9]. However, the concentration of Hg in this area is still relatively high, and it is necessary to carry out continuous observations of different forms of atmospheric Hg in the largest cities in Northwestern China to find out its long-term trend and main controlling factors.

Hg has long been perceived as a global toxic pollutant due to its bioaccumulation and is a hazard to public health [10]. Hg is known to have neurotoxic and teratogenic effects. Humans are usually exposed to Hg in a chronic and low-dose fashion [11]. Excessive amounts of Hg may lead to various health problems, such as corrosive bronchitis and interstitial pneumonia, and may damage the brain, lung, and kidney tissues of those exposed, as well as damaging the central nervous system through the blood [11,12]. GEM is mainly (~80%) absorbed in human lungs [13] and can spread throughout the body, cross the blood–brain barrier, and accumulate in the central nervous system [14]. Research also shows that the level of GOM absorption in the human body caused by inhalation is approximately 50% lower than that of GEM. However, absorption by ingestion is higher than that of GEM (0.01%), in the range of 7–14% [13].

Xi’an, the capital of Shaanxi Province, is the largest city in northwest China and is experiencing severe air pollution due to the unfavorable terrain and diffusion conditions, as well as the reliance on coal [15]. It is listed as a key area for air pollution control in China, along with the Beijing–Tianjin–Hebei region and the Yangtze River Delta region. However, there is limited research on the atmospheric Hg pollution in the region. In a previous study, our group documented 3 years (2009−2012) of variations in total gaseous mercury (TGM) concentrations in Xi’an [9], but no research has been conducted on GEM yet. In addition, some policies on controlling atmospheric mercury have been released in recent years, such as the Minamata Convention on Hg that went into force in 2017 [16]. Therefore, high-resolution and relatively long-term observations of GEM and GOM concentrations in the atmosphere of Xi’an are necessary, aiming to understand the seasonal and diurnal variations, filling the data blank, and estimating the contribution of coal combustion reduction to Hg pollution. We also assessed the non-carcinogenic health risks of atmospheric Hg and determined the health impacts of atmospheric Hg for local residents to clarify the health benefits of reduced Hg concentrations.

## 2. Materials and Methods

### 2.1. Sampling Site

**Figure 1:**
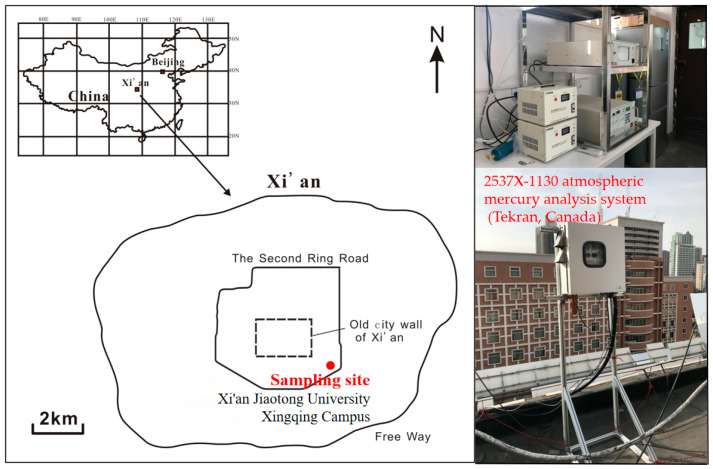
The location of the sampling site and Hg sampling system.

Xi’an (34°14′ N, 108°59′ E) is the largest city in Northwestern China and the capital of Shaanxi Province, with an area of about 10,000 km^2^ and a population of 10 million. It is located at an elevation of 400 m above sea level and situated between the Yellow River Valley and the center of the Guanzhong Plain. The city borders the northern foot of the Qin Mountain to the south and the banks of the Wei River to the north. Xi’an has a warm, temperate, semi-humid continental monsoon climate, with cold and dry winters (divided into 16 November to 15 March each year based on the domestic heating period), warm and dry springs (16 March to 31 May), hot and humid summers (1 June to 31 August), and cool and pleasant autumns (1 September to 15 November). The monitoring site of this study is located on the rooftop of the 5th floor of the teaching building in the second district of Xingqing Campus of Xi’an Jiaotong University (Figure 1), which belongs to a mixed area of transportation, residents, and education.

### 2.2. Measurements of Speciated Mercury

The concentrations of GEM and GOM in the atmosphere were continuously measured using a 2537X-1130 atmospheric mercury analysis system (Tekran, Toronto, ON, Canada) from 1 April 2019 to 31 December 2020. During the sampling, the ambient air first enters the coarse particle impactor to remove particles above PM_10_ and then passes through the 1130 sampling unit. In this unit, GOM is captured by the KCl coating inside the annular diffusion tube, and then air mass directly enters the 2537X mercury analyzer to measure GEM. After a sampling process is completed, the system enters the analysis process. After zero gas blowing and purification, the annular diffusion tube of the 1130 sampling unit is heated to analyze GOM. GOM undergoes high-temperature cracking to form GEM, which is then loaded into a 2537X analyzer with zero gas, and the GOM content is obtained through calculation.

The Tekran atmospheric mercury analyzer has a built-in mercury permeation source that can automatically calibrate the instrument at regular intervals (every hour). It can also be manually calibrated and checked by injecting mercury vapor through the analyzer injection port. The 1130D sample gas filtration membrane and coarse particle filter are replaced every two weeks. The resolution of the GEM and GOM online data obtained in this study is 5 min and 1 h, respectively. The GEM data have a 20 min gap every hour for instrument calibration. In the whole sampling period in this study, the percentage of missing GEM data was 39.6%, of which 33.3% was due to routine calibration and 6.3% was due to technical maintenance and power outage. The percentage of missing GOM data was 25.6% due to technical maintenance and power outage.

### 2.3. Health Risk Assessment

The health risk assessment model, created by the United States Environmental Protection Agency [17], is used here to determine health risk of GEM in the air at the site. The chronic exposure concentration (CEC) from inhaling GEM is calculated using the following equation:

CEC_Hg_ = C_GEM_ × IR × ET × EF × ED/(BW × AT), (1)

where C_GEM_ is the concentration of GEM (ng/m^3^); IR is the inhalation rate (m^3^/d); exposure time (ET) = 24 h/day; exposure frequency (EF) = 365 day/year; exposure duration (ED) = age of the exposed person; BW is body weight (kg); average time of exposure (AT) = ED × 365 day; and the IR and BW parameters are shown in Table S1.

The assessment of non-carcinogenic health risk is called the hazard quotient (HQ). It is calculated by dividing the normalization of a certain chemical exposure by a specific reference dose, shown as Equation (2):

HQ = CEC_Hg_/RFC_Hg_, (2)

where RfC is the amount of Hg that is considered safe to breathe in a reference dose for inhalation exposure (3 × 10^−4^ mg/m^3^) as recommended by the USEPA [18]. When the HQ value is less than 1, it means that no harmful effects on health or non-carcinogenic adverse effects are indicated [19].

### 2.4. Data Collection and Statistical Analysis

This study obtained the hourly average concentrations of the air pollutants PM_10_, PM_2.5_, SO_2_, NO_2_, CO, and O_3_ during the observation period through the PM_2.5_ Historical Data Network [20] and obtained daily meteorological factors such as temperature (T), wind speed (WS), relative humidity (RH), and visibility (VISIB) data from the Xi’an Meteorological Bureau [21].

Other pollutant data, including organic carbon (OC), elemental carbon (EC), and elements in PM_2.5_ used in this study were from the Institute of Earth Environment, Chinese Academy of Sciences (34°13′ N, 109°0′ E). The daily PM_2.5_ filter samplers were deployed approximately 10 m above ground level using mini-volume air samplers (Airmetrics, Eugene, OR, USA) that operated at a flow rate of 5 L min^−1^. A Desert Research Institute (DRI) Model 2001 Thermal/Optical Carbon Analyzer (Atmoslytic Inc., Calabasas, CA, USA) was used for carbon analysis following the IMPROVE_A (Interagency Monitoring of Protected Visual Environment) thermal/optical reflectance protocol [22]. Energy Dispersive X-Ray Fluorescence (ED-XRF) spectrometry (Epsilon 5 ED-XRF, PANalytical B. V., Almelo, The Netherlands) was used to determine the concentrations of elements. The specific sampling and analysis methods can be found in our previous research [23].

This study used the *t*-test method in SPSS Statistics 27 to analyze whether meteorological factors affected changes in mercury concentration during the observation period. The Mann–Kendall trend test in Excel Macro was used to quantify the monthly mean of atmospheric mercury and the decreasing trend of non-carcinogenic health risks during the observation period.

## 3. Results

### 3.1. Variation in GEM and GOM in Atmosphere

**Figure 2:**
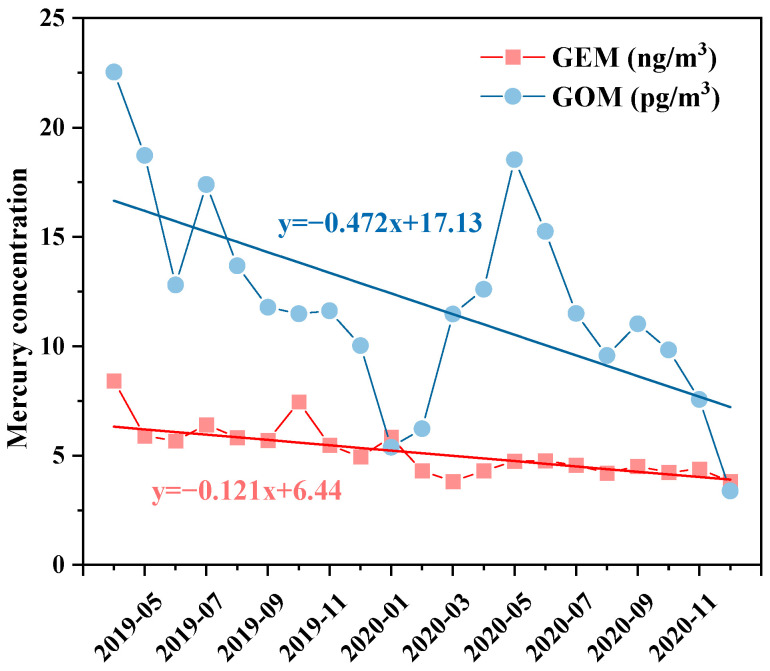
The average monthly variation in GEM and GOM concentrations and Sen’s regression curves.

The average and standard deviation values of the GEM and GOM concentrations were 5.78 ± 7.36 ng/m^3^ and 14.2 ± 20.8 pg/m^3^, respectively, with ranges of 0.022–297 ng/m^3^ and 0.092–381 pg/m^3^. It can be seen that the fluctuation in the concentrations was very large, and the concentration level of GEM was 2–3 orders of magnitude higher than that of GOM (Figure S1). The Mann–Kendall trend test (Figure 2) was used to quantitatively study the decreasing trend of the atmospheric monthly average Hg concentration. The Sen’s slope of the GEM monthly concentration was −0.121 (Mann–Kendall trend test *p* < 0.0001), and the Sen’s slope of the GOM monthly concentration was −0.472 (Mann–Kendall trend test *p* < 0.0001). It can be seen that from 1 April 2019 to 31 December 2020, the concentration of GEM in Xi’an decreased at a rate of 0.121 ng/m^3^ per month, and the decreasing trend of GOM was more pronounced, with a rate of 0.472 pg/m^3^ per month.

The average concentrations of GEM and GOM also decreased from 6.13 ng/m^3^ and 14.29 pg/m^3^ in 2019 to 4.46 ng/m^3^ and 10.53 pg/m^3^ in 2020, respectively. The downward trend may be related to the increasing environmental supervision efforts in Xi’an since 2019: the reduction in or even closure of some high-energy-consuming and high-polluting enterprises, the decrease in thermal power generation of power production enterprises, and the “coal to clean energy” work carried out by non-power enterprises [24]. According to the Xi’an Statistical Yearbook [21], it can be seen that the raw coal consumption of industrial enterprises above designated size in Xi’an was 1.03 million tons to 0.909 million tons from 2019 to 2020, also showing a downward trend. A *t*-test was conducted on the annual average concentrations of GEM and GOM, and it was found that there was a significant difference of *p* < 0.01 in the concentration values between the two years. This indicates that the decreasing trend of GEM and GOM concentrations year by year was significant. A *t*-test was also conducted on the annual average meteorological factors over the past two years (Figure S2); except for temperature, no significant changes were observed in other meteorological factors. It can be seen that the annual decrease in atmospheric Hg concentration during the observation period was less affected by meteorological factors. The reduction in coal consumption and the resulting decrease in atmospheric pollutant emissions may be the dominant factors contributing to the decrease in atmospheric Hg concentration in Xi’an. This will be further demonstrated in the correlation analysis in Section 3.3.2.

Table 1 summarizes previous studies of atmospheric Hg worldwide. The concentration of GEM in the atmosphere of Xi’an was higher than that of all regions except Guiyang (9.72 ± 10.2 ng/m^3^). Compared with other regions except Guiyang the atmospheric GEM concentration in Xi’an was 1.8–6.72 times higher, with the lowest concentration observed from October 2012 to July 2017 in Patagonia (Argentina), within the Southern Volcanic Zone of South America, with a concentration of 0.860 ± 0.160 ng/m^3^ [25]. The concentration of GOM in the atmosphere of Xi’an was lower than Guiyang (35.7 ± 43.9 pg/m^3^), Xiamen (61.1 pg/m^3^), Chicago (17.0 ± 87.0 pg/m^3^), and the Pic Du Midi Observatory in France (27 ± 34 pg/m^3^). Compared with other regions except these four areas, the atmospheric GOM concentration in Xi’an was 1.17–8.35 times higher, with the lowest concentration observed from October 2009 to December 2018 in Okinawa, with a concentration of 1.70 ± 2.90 pg/m^3^ [26]. The higher concentrations of GEM and GOM in Guiyang might be due to the elevated anthropogenic emissions such as residential coal burning [27], similar to Xi’an.

### 3.2. Diurnal Variations of Hg Species

**Figure 3:**
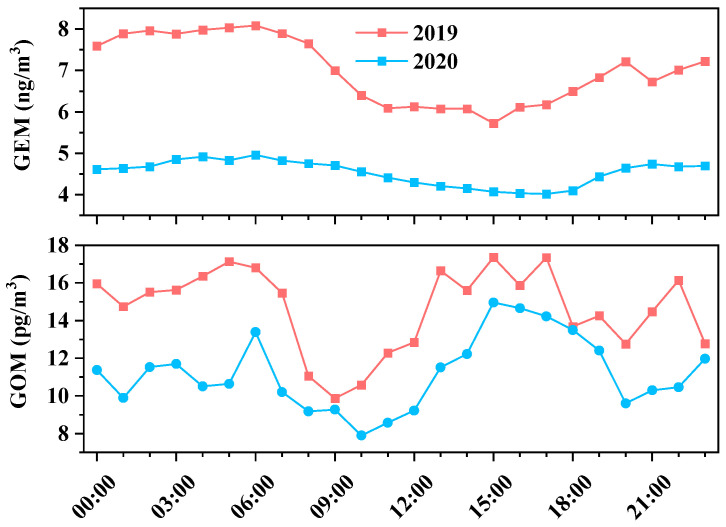
Diurnal variation of GEM and GOM concentrations in 2019 and 2020.

Figure 3 shows a comparison of the diurnal variations in GEM and GOM concentrations in Xi’an, and the data are the hourly average observed concentration. Over the past two years, the diurnal variation trend of atmospheric Hg has been relatively similar. This is consistent with previous research in China [3,25,27,28], which showed that the average concentration of GEM at night is always higher than during the day. This is mainly related to the height of the atmospheric boundary layer, which is lower at night and makes it difficult for pollutants to diffuse, leading to the accumulation of air pollutants [28]. The drastic diurnal changes in 2019 compared to 2020, namely higher nighttime concentrations, may also be contributed by nighttime emission sources, which were weakened in 2020.

The average concentration of GOM in this study showed two peaks within a day, exhibiting an M-shaped variation pattern. The two peaks occurred around 5:00–8:00 in the morning and 13:00–19:00 in the afternoon, almost consistent with the daily commuting time. Previous research has found a possible correlation between the increase in GOM concentration and emissions of motor vehicle fuel combustion [40]. In addition, after 9:00 am, solar radiation gradually increases, and GEM undergoes an oxidation reaction under the action of oxidants such as O_3_, generating GOM [3]. Therefore, the concentration of GOM showed an upward trend after 9:00 am. Therefore, under the combined influence of anthropogenic sources (especially automotive exhaust emission) and atmospheric photochemical reactions, the concentration of GOM shows a fluctuating trend within a day.

### 3.3. Seasonal Variations and Influence Factor of Hg Species

#### 3.3.1. Seasonal Variations of Hg Species

**Figure 4:**
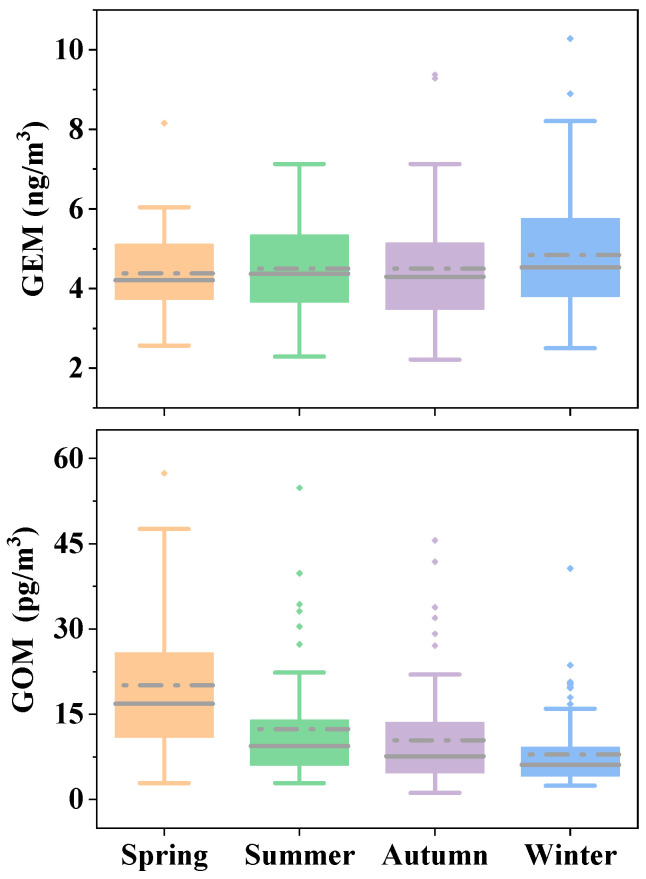
Seasonal variations in GEM and GOM concentrations from November 2019 to November 2020. The dotted gray lines represent the mean values, the solid gray lines within each box represent the median values, the boundaries of the boxes represent 25th and 75th percentiles, the whiskers indicate 10th and 90th percentiles, and the small dots represent outliers.

Figure 4 shows the seasonal variations (mean of observed high-resolution concentration data according to season) of GEM and GOM concentrations from 16 November 2019 to 15 November 2020, the four seasons during this study. The mean concentration of GEM in winter was the highest (4.84 ± 1.45 pg/m^3^), and the concentrations were comparable in autumn (4.51 ± 1.37 pg/m^3^), summer (4.51 ± 1.14 pg/m^3^), and spring (4.38 ± 1.12 pg/m^3^), consistent with previous studies [41]. The atmospheric mixing height and the wind speed were both low in winter during the selected period; for example, the average wind speeds of spring, summer, autumn, and winter were 5.05, 5.43, 4.67, and 4.25 m/s, respectively, causing difficult diffusion of air pollutants in winter. In addition to weather conditions, the increase in coal consumption during the heating season also can result in a higher concentration of GEM in winter [42,43]. The high concentration of GEM in winter is also due to the dominant wind direction in Xi’an being northeast wind. Hancheng City and Shanxi Province in the northeast direction of Xi’an are coal industrial bases with huge coal production and consumption. The total coal consumption in Shanxi Province in 2019 and 2020 was 34,900 and 36,200 million tons, respectively [44], which had a serious impact on the atmospheric environment of Xi’an located in the downwind direction.

GOM showed more distinct seasonality in this study. The mean concentration of GOM was the highest in spring (20.09 ± 13.09 pg/m^3^), followed by summer (12.39 ± 9.70 pg/m^3^), autumn (10.45 ± 9.14 pg/m^3^), and winter (7.94 ± 5.66 pg/m^3^), mainly because of the complex chemical behavior of GOM in the atmosphere [45]. The high levels of GOM were also found in urban areas of New York State, Xiamen, and Beijing during the spring [33,46]. GOM can be formed by photochemical processes involving the oxidation of GEM. The seasonal variations in GOM may be partly ascribed to the photochemical oxidation processes, which are more active in spring and summer than in other seasons because of increased solar radiation, ozone, and other atmospheric oxidants [47]. Studies have observed that the concentration of GOM on non-rainy summer days is much higher than on rainy days, indicating that rain can significantly remove GOM from the atmosphere [48]. According to the *Xi’an Statistical Yearbook* [21], from March to May 2020, Xi’an received a total of 12 rainfall events, while from June to August, it received 41 rainfall events. Therefore, abundant precipitation is the main reason for the lower GOM concentration in Xi’an in summer compared to spring. The highest GOM in spring in the current study is the result of the balance between the generation of photochemical reactions caused by solar radiation and the clearance of GOM caused by precipitation.

#### 3.3.2. Influence Factor of Hg in Different Seasons: Further Evidence of Hg Reduction

**Figure 5:**
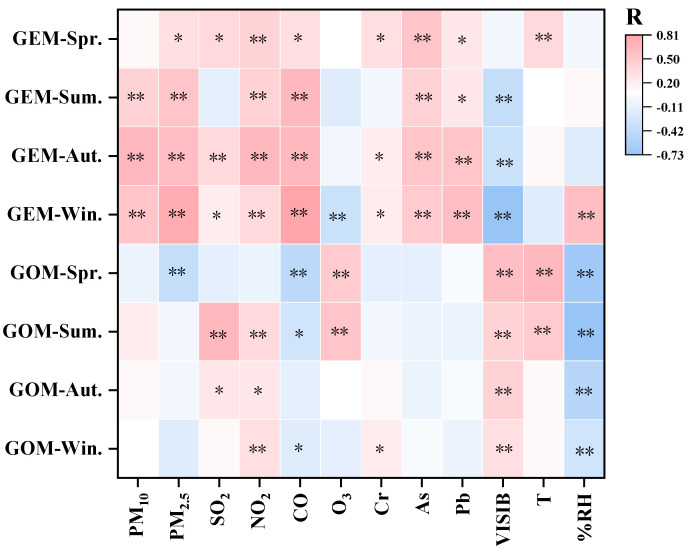
Correlation between GEM, GOM, and other air pollutants (Cr, As, and Pb refer to their concentrations in PM_2.5_) and meteorological factors; ** indicates significant correlation at the 0.01 level (double tailed); * indicates significant correlation at the 0.05 level (double tailed).

To explain the influence factors of Hg seasonal variations and the contribution of fossil fuels (coal burning) to Hg reductions, this study conducted a correlation analysis between GEM and GOM in four seasons and studied the visibility, temperature, relative humidity, and concentrations of air pollutants (SO_2_, NO_2_, CO, O_3_, PM_2.5_, and PM_10_) from 16 November 2019 to 15 November 2020. As shown in Figure 5, the concentrations of GEM was significantly positively correlated with the concentrations of PM_10_ (*p* < 0.01 in summer, autumn, and winter), PM_2.5_ (*p* < 0.01 in summer, autumn, and winter and <0.05 in spring), SO_2_ (*p* < 0.01 in autumn and <0.05 in spring and no significant correlation observed in summer when coal consumption is low), NO_2_ (*p* < 0.01 in all seasons), and CO (*p* < 0.01 in summer, autumn, and winter and <0.05 in spring). Similar to Hg, SO_2_, NO_2_, and CO in the atmosphere mainly come from the combustion of fossil fuels such as coal, and the PM_2.5_ concentration is also affected by the burning of fossil fuels [49]. The high correlation coefficient between GEM and the above air pollutants provides evidence for the significant contribution of fossil fuel combustion to atmospheric GEM.

Moreover, a correlation analysis was also conducted between OC, EC, and elements in PM_2.5_ and atmospheric mercury (GEM and GOM) during the observation period, and the results are shown in Table S2. Among them, Cr, As, and Pb have a good correlation with GEM, and these three heavy metals have been widely proven to be closely positively correlated with emissions from coal combustion [50,51,52]. Further dividing the seasons, the concentration of GEM significantly positively correlated with Cr in spring, autumn, and winter (*p* < 0.05), As in all seasons (*p* < 0.01), and Pb in autumn and winter (*p* < 0.01) and spring and summer (*p* < 0.05). We also noticed that the concentrations of As, Pb, and Cr significantly decreased during the sampling period of this study (Figure S3), with monthly decreasing rates of 0.172, 0.110, and 0.165 ng/m^3^, respectively. This is basically consistent with the decline in GEM.

The concentration of GEM was almost negatively correlated with visibility year round. There may be two reasons for this phenomenon: (1) The decrease in visibility, in addition to the effects of natural phenomena such as blizzards, sandstorms, etc., is usually caused by hazy days [53]. The haze in Xi’an is mainly caused by high concentrations of PM_2.5_, which means an increase in the intensity of anthropogenic emissions, which may lead to an increase in the GEM concentration. This also precisely explains the positive correlation between PM_2.5_ and GEM. (2) The meteorological conditions during hazy periods are usually unfavorable for the diffusion of air pollutants and are prone to temperature inversion. Adverse meteorological conditions such as the atmospheric inversion layer may also lead to an increase in atmospheric GEM concentration [54].

This study found a positive correlation between GOM and O_3_, especially in spring (*p* < 0.01) and summer (*p* < 0.05). The correlation between GOM and O_3_ is complex and influenced by multiple factors. In the atmosphere, the oxidation process of Hg can convert GEM into GOM, which may be related to O_3_, which may be due to O_3’_s involvement as an oxidant in the conversion process of GEM to GOM [55]. In addition, GOM showed a significant positive correlation with visibility and a significant negative correlation with RH. The increase in RH can promote the conversion of GOM to PBM [56]. When the relative humidity increases, the water vapor content in the air increases, and aerosol particles may absorb moisture, leading to a decrease in visibility [57]; therefore, GOM is significantly positively correlated with visibility and negatively with RH.

### 3.4. Human Health Risk Assessment

**Figure 6:**
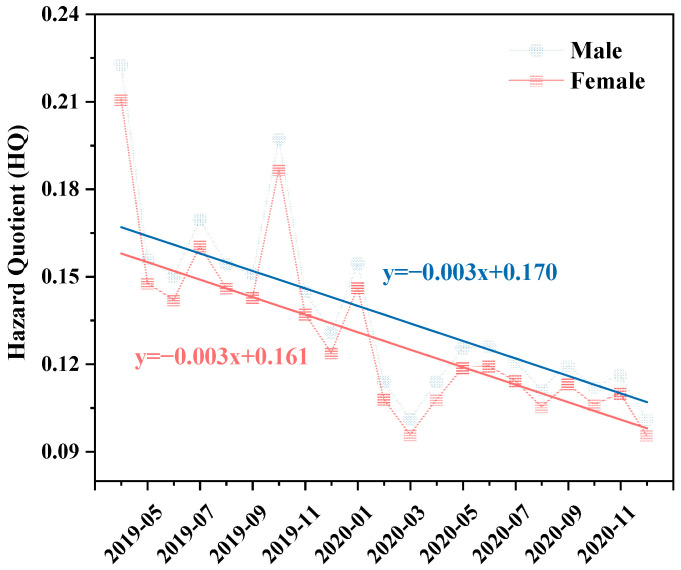
The hazard quotient (HQ) and Sen’s regression curves of GEM for different genders in Xi’an during the study period.

According to the USEPA model for non-carcinogenic risks of GEM to human health, we calculated the hazard quotient (HQ), as shown in Figure 6, which shows the inhalable health risk of GEM for males and females based on the monthly concentrations of GEM. The Mann–Kendall trend test was also used to quantitatively study the decreasing trend of HQ. The Sen’s slopes of males and females were both −0.003 (Mann–Kendall trend test *p* < 0.0001), indicating that the HQ of GEM for both genders decreased at a rate of 0.003 per month from 1 April 2019 to 31 December 2020. A *t*-test was conducted on the HQ in males and females, and no significant changes were found. In this study, the HQ was far less than 1, meaning that GEM does not pose a significant non-carcinogenic risk to local residents. However, due to the bioaccumulations of Hg, the long-term exposure to low dosages of Hg would be harmful to human health [1], such as mercury poisoning nephrotic syndrome and peripheral neuropathy [58]. Therefore, the health risks of GEM should still be taken seriously. Establishing long-term monitoring measures helps account for pollution loads and helps planning to restore healthy environmental conditions.

**Figure 7:**
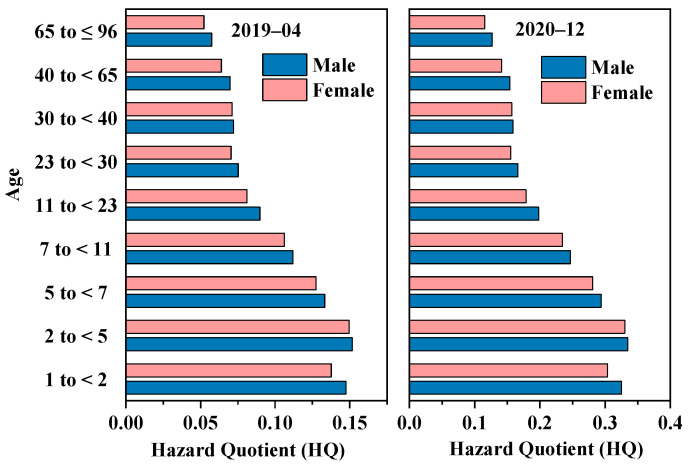
Comparison of HQ of GEM at different age groups and different genders in April 2019 and December 2020 in Xi’an.

April 2019 and December 2020 were the two months with the highest and lowest GEM concentrations in Xi’an during the study period, respectively. The concentration of GEM decreased from 8.42 to 3.82 ng/m^3^ between these two months, with a decrease of 54.7%. Correspondingly, the HQ of GEM also decreased by 54.7%. We divided the exposed population by gender and nine age groups, covering ages 1 to 96 (Figure 7), and compared the HQ of GEM between April 2019 and December 2020. Overall, the distribution pattern of the non-carcinogenic risk of GEM across age and gender was completely similar at two months. Regardless of gender, GEM posed the highest inhalation health risk to children aged 2–5, followed by infants aged 1–2, and then the risk decreased with age in all other groups. The HQ of GEM for 2–5-year-old children was 2.57 and 2.63 times higher than that of 65–96-year-old males and females (the lowest risk group), respectively, indicating that children were more sensitive to non-carcinogenic risks and should hence minimize their exposure to GEM. Among the same age groups, GEM always posed a higher health risk to men than to women, indicating that men are more sensitive to the non-carcinogenic risk of GEM.

## 4. Conclusions

The mean concentrations of GEM and GOM in the atmosphere of Xi’an City from 1 April 2019 to 31 December 2020 were 5.78 ± 7.36 ng/m^3^ and 14.2 ± 20.8 pg/m^3^, respectively. The concentration of GEM decreased at a rate of 0.121 ng/m^3^ per month, and GOM was at a rate of 0.472 pg/m^3^ per month. This downward trend is more affected by the decrease in coal consumption than meteorological factors. The concentration of GEM in winter was the highest, controlled by primary emissions and adverse meteorological factors, while the concentration of GOM in spring was the highest, mainly dominated by the photochemical oxidation processes. GEM was positively correlated with SO_2_, NO_2_, CO, PM_2.5_, PM_10_, and Cr, As, and Pb in PM_2.5_ and negatively correlated with visibility, further evidence of the contribution of coal combustion to GEM. GOM was positively correlated with O_3_ and visibility but negatively correlated with RH. The health risk assessment indicates that the HQ of GEM for both males and females decreased at a rate of 0.003 per month during the study period. GEM posed the highest inhalation risk to children aged 2–5, and always posed a higher health risk to males than females. The results of this study identified the main sources, controlling factors, and key protected populations of atmospheric Hg pollution in northern China, providing scientific recommendations for improving the local environment and protecting populations.

## Figures and Tables

**Table 1:** Comparison of GEM and GOM monitored at various locations worldwide.

Location	Sampling Period	GEM (ng/m^3^)	GOM (pg/m^3^)	Reference
Xi’an, China	April 2019–December 2020	5.78 ± 7.36	14.2 ± 20.8	This study
Guiyang, China	August–December 2009	9.72 ± 10.2	35.7 ± 43.9	[27]
Beijing, China	December 2008–November 2009	3.22 ± 1.74	10.1 ± 18.8	[28]
Xiamen, China	March 2012–February 2013	3.50	61.1	[29]
Taoyuan, Taiwan	October 2017–September 2018	2.61 ± 6.47	12.1 ± 34.3	[30]
Gyodong Island, Korea	August 2015–September 2017	2.80 ± 2.90	3.60 ± 3.50	[26]
Patagonia, Argentina	October 2012–July 2017	0.860 ± 0.160	/	[25]
	March 2014–July 2017	/	4.61 ± 4.00	
Mumbai, India	January–December 2017	3.10 ± 1.10	/	[31]
Chicago, USA	July–November 2007	2.50 ± 1.50	17.0 ± 87.0	[32]
Rochester, USA	December 2007–November 2009	1.60 ± 0.400	5.30 ± 10.7	[33]
Beltsville, USA	2007–2015	1.41 ± 0.230	4.60 ± 33.7	[34]
HCMC, Vietnam	July–October 2022	1.61 ± 0.32	/	[35]
Okinawa, Japan	October 2009–December 2018	1.81 ± 0.43	1.70 ± 2.90	[36]
Pic Du Midi Observatory, France	November 2011–November 2012	1.86 ± 0.27	27.0 ± 34.0	[37]
Dartmouth, Canada	January 2010–December 2011	1.67 ± 1.01	2.10 ± 3.40	[38]
North Atlantic Ocean	2003	1.63 ± 0.08	5.9 ± 4.9	[39]

## Data Availability

Data will be made available on request.

## Supplementary Materials

The following supporting information can be downloaded at: https://www.mdpi.com/article/10.3390/toxics12120935/s1; Table S1: Distribution percentiles of physiological daily inhalation rates (m^3^/day) for free-living normal-weight males and females aged 1 to 96 years; Table S2: Correlation between GEM, GOM, and carbonaceous aerosols, elements in PM_2.5_; Figure S1: The daily variation in GEM and GOM concentrations from April 2019 to December 2020 in Xi’an; Figure S2: The daily variation in meteorological factors from April 2019 to December 2020 in Xi’an; Figure S3: The average monthly variation in heavy metal (As, Pb, and Cr) concentrations and GEM during the sampling period.

## References

[1] Wang Z., Zhang Y., Wang L., Li X., Zhou X., Li X., Yan M., Lu Q., Tang Z., Zhang G. (2021). Characteristics and Risk Assessments of Mercury Pollution Levels at Domestic Garbage Collection Points Distributed within the Main Urban Areas of Changchun City. Toxics.

[2] Yin X., Zhou W., Kang S., de Foy B., Yu Y., Xie J., Sun S., Wu K., Zhang Q. (2020). Latest observations of total gaseous mercury in a megacity (Lanzhou) in northwest China. Sci. Total Environ..

[3] Duan L., Wang X., Wang D., Duan Y., Cheng N., Xiu G. (2017). Atmospheric mercury speciation in Shanghai, China. Sci. Total Environ..

[4] Zhao Y., Mann M.D., Olson E.S., Pavlish J.H., Dunham G.E. (2006). Effects of sulfur dioxide and nitric oxide on mercury oxidation and reduction under homogeneous conditions. J. Air Waste Manag. Assoc..

[5] Edwards B.A., Kushner D.S., Outridge P.M., Wang F. (2021). Fifty years of volcanic mercury emission research: Knowledge gaps and future directions. Sci. Total Environ..

[6] Pirrone N., Cinnirella S., Feng X., Finkelman R.B., Friedli H.R., Leaner J., Mason R., Mukherjee A.B., Stracher G.B., Streets D.G. (2010). Global mercury emissions to the atmosphere from anthropogenic and natural sources. Atmos. Chem. Phys..

[7] Sherman L.S., Blum J.D., Keeler G.J., Demers J.D., Dvonch J.T. (2011). Investigation of Local Mercury Deposition from a Coal-Fired Power Plant Using Mercury Isotopes. Environ. Sci. Technol..

[8] Xu H., Cao J., Chow J.C., Huang R.J., Shen Z., Chen L.W.A., Ho K.F., Watson J.G. (2016). Inter-annual variability of wintertime PM 2.5 chemical composition in Xi’an, China: Evidences of changing source emissions. Sci. Total Environ..

[9] Xu H., Sonke J.E., Guinot B., Fu X., Sun R., Lanzanova A., Candaudap F., Shen Z., Cao J. (2017). Seasonal and Annual Variations in Atmospheric Hg and Pb Isotopes in Xi’an, China. Environ. Sci. Technol..

[10] Driscoll C.T., Mason R.P., Chan H.M., Jacob D.J., Pirrone N. (2013). Mercury as a Global Pollutant: Sources, Pathways, and Effects. Environ. Sci. Technol..

[11] Singh S., Kumar V. (2019). Mercury detoxification by absorption, mercuric ion reductase, and exopolysaccharides: A comprehensive study. Environ. Sci. Pollut. Res..

[12] Niane B., Devarajan N., Poté J., Moritz R. (2019). Quantification and characterization of mercury resistant bacteria in sediments contaminated by artisanal small-scale gold mining activities, Kedougou region, Senegal. J. Geochem. Explor..

[13] Jung-Duck P., Wei Z. (2012). Human exposure and health effects of inorganic and elemental mercury. J. Prev. Med. Public Health = Yebang Uihakhoe Chi.

[14] Bjørklund G., Dadar M., Mutter J., Aaseth J. (2017). The toxicology of mercury: Current research and emerging trends. Environ. Res..

[15] Dai Q., Bi X., Song W., Li T., Liu B., Ding J., Xu J., Song C., Yang N., Schulze B.C. (2019). Residential coal combustion as a source of primary sulfate in Xi’an, China. Atmos. Environ..

[16] Feng X., Fu X., Zhang H., Wang X., Jia L., Zhang L., Lin C.-J., Huang J.-H., Liu K., Wang S. (2024). Combating air pollution significantly reduced air mercury concentrations in China. Natl. Sci. Rev..

[17] USEPA (2009). Risk Assessment Guidance for Superfund Volume I: Human Health Evaluation Manual.

[18] USEPA (2020). Regional Screening Levels (RSLs)—Resident Ambient Air Table (TR¼1E-06, HQ¼1).

[19] USEPA (2001). Methods for Collection, Storage and Manipulation of Sediments for Chemical and Toxicological Analyses.

[20] Air Quality Index Historical Data Historical Data on PM_2.5_. https://www.aqistudy.cn/historydata/.

[21] Xi’an Municipal Bureau of Statistics Xi’an Statistical Yearbook. http://tjj.xa.gov.cn/.

[22] Chow J.C., Watson J.G., Chen L.W.A., Chang M.C.O., Robinson N.F., Trimble D., Kohl S. (2012). The IMPROVE_A Temperature Protocol for Thermal/Optical Carbon Analysis: Maintaining Consistency with a Long-Term Database. J. Air Waste Manag. Assoc..

[23] Xu H.M., Cao J.J., Ho K.F., Ding H., Han Y.M., Wang G.H., Chow J.C., Watson J.G., Khol S.D., Qiang J. (2012). Lead concentrations in fine particulate matter after the phasing out of leaded gasoline in Xi’an, China. Atmos. Environ..

[24] Shaanxi Provincial Bureau of Statistics Xi’an: “Coal to Clean” Has Achieved Significant Results in Improving Energy Efficiency in Large-Scale Industries. http://tjj.shaanxi.gov.cn/ggbf/sxxx/202004/t20200413_1626597.html.

[25] Diéguez M.C., Bencardino M., García P.E., D’Amore F., Castagna J., De Simone F., Soto Cárdenas C., Ribeiro Guevara S., Pirrone N., Sprovieri F. (2019). A multi-year record of atmospheric mercury species at a background mountain station in Andean Patagonia (Argentina): Temporal trends and meteorological influence. Atmos. Environ..

[26] Lee S.-H., Lee J.-I., Kim P.-R., Kim D.-Y., Jeon J.-W., Han Y.-J. (2019). Factors influencing concentrations of atmospheric speciated mercury measured at the farthest island West of South Korea. Atmos. Environ..

[27] Fu X., Feng X., Qiu G., Shang L., Zhang H. (2011). Speciated atmospheric mercury and its potential source in Guiyang, China. Atmos. Environ..

[28] Zhang L., Wang S.X., Wang L., Hao J.M. (2013). Atmospheric mercury concentration and chemical speciation at a rural site in Beijing, China: Implications of mercury emission sources. Atmos. Chem. Phys..

[29] Xu L., Chen J., Yang L., Niu Z., Tong L., Yin L., Chen Y. (2015). Characteristics and sources of atmospheric mercury speciation in a coastal city, Xiamen, China. Chemosphere.

[30] Sheu G.-R., Phu Nguyen L.S., Truong M.T., Lin D.-W. (2019). Characteristics of atmospheric mercury at a suburban site in northern Taiwan and influence of trans-boundary haze events. Atmos. Environ..

[31] Rao M.N., Latha R., Nikhil K., Murthy B.S. (2024). Atmospheric gaseous mercury and associated health risk assessment in the economic capital of India. Environ. Monit. Assess..

[32] Gratz L.E., Keeler G.J., Marsik F.J., Barres J.A., Dvonch J.T. (2013). Atmospheric transport of speciated mercury across southern Lake Michigan: Influence from emission sources in the Chicago/Gary urban area. Sci. Total Environ..

[33] Choi H.-D., Huang J., Mondal S., Holsen T.M. (2013). Variation in concentrations of three mercury (Hg) forms at a rural and a suburban site in New York State. Sci. Total Environ..

[34] Ren X., Luke W.T., Kelley P., Cohen M.D., Artz R., Olson M.L., Schmeltz D., Puchalski M., Goldberg D.L., Ring A. (2016). Atmospheric mercury measurements at a suburban site in the Mid-Atlantic United States: Inter-annual, seasonal and diurnal variations and source-receptor relationships. Atmos. Environ..

[35] Nguyen L.S.P., Hien T.T. (2023). Long-Range Atmospheric Mercury Transport from Across East Asia to a Suburban Coastal Area in Southern Vietnam. Bull. Environ. Contam. Toxicol..

[36] Marumoto K., Suzuki N., Shibata Y., Takeuchi A., Takami A., Fukuzaki N., Kawamoto K., Mizohata A., Kato S., Yamamoto T. (2019). Long-Term Observation of Atmospheric Speciated Mercury during 2007–2018 at Cape Hedo, Okinawa, Japan. Atmosphere.

[37] Fu X., Marusczak N., Heimbürger L.-E., Sauvage B., Gheusi F., Prestbo E.M., Sonke J.E. (2016). Atmospheric mercury speciation dynamics at the high-altitude Pic du Midi Observatory, southern France. Atmos. Chem. Phys..

[38] Cheng I., Zhang L., Mao H., Blanchard P., Tordon R., Dalziel J. (2014). Seasonal and diurnal patterns of speciated atmospheric mercury at a coastal-rural and a coastal-urban site. Atmos. Environ..

[39] Laurier F., Mason R. (2007). Mercury concentration and speciation in the coastal and open ocean boundary layer. J. Geophys. Res. Atmos..

[40] Lu R., Wu Y., Zhang X., Shen Y., Wu F., Xue Y., Zou Q., Ma C. (2020). Distribution Characteristics and Source Analysis of Mercury Forms in the Atmosphere of Suzhou City. Environ. Sci..

[41] Xu Z., Chen L., Zhang Y., Han G., Chen Q., Chu Z., Zhang Y., Li C., Yang Y., Wang X. (2022). Meteorological Drivers of Atmospheric Mercury Seasonality in the Temperate Northern Hemisphere. Geophys. Res. Lett..

[42] Liu J., Wang L., Zhu Y., Lin C.-J., Jang C., Wang S., Xing J., Yu B., Xu H., Pan Y. (2019). Source attribution for mercury deposition with an updated atmospheric mercury emission inventory in the Pearl River Delta Region, China. Front. Environ. Sci. Eng..

[43] Wang X., Lin C.J., Feng X., Yuan W., Fu X., Zhang H., Wu Q., Wang S. (2018). Assessment of Regional Mercury Deposition and Emission Outflow in China’s mainland. J. Geophys. Res. Atmos..

[44] Shanxi Municipal Bureau of Statistics Shanxi Statistical Yearbook. https://tjj.shanxi.gov.cn/.

[45] Barago N., Floreani F., Acquavita A., Esbrí J.M., Covelli S., Higueras P. (2020). Spatial and Temporal Trends of Gaseous Elemental Mercury over a Highly Impacted Coastal Environment (Northern Adriatic, Italy). Atmosphere.

[46] Zhang H., Wang Z., Wang C., Zhang X. (2019). Concentrations and gas-particle partitioning of atmospheric reactive mercury at an urban site in Beijing, China. Environ. Pollut..

[47] Si L., Ariya P. (2018). Recent Advances in Atmospheric Chemistry of Mercury. Atmosphere.

[48] Maximilian K.A., Olivier M., Paolo L., Marcos A., Isabel M., Fernando V., Grover S., René G., Luis B., Diego A. (2021). Seasonal patterns of atmospheric mercury in tropical South America as inferred by a continuous total gaseous mercury record at Chacaltaya station (5240 m) in Bolivia. Atmos. Chem. Phys..

[49] Lin C.-C., Macrohon J.K.E., Brimblecombe P., Adyanis L.N., Yeh C.-F., Lai C.-H., Wang L.-C. (2023). Atmospheric mercury speciation and concentration at the urban and industrial sites in Taiwan over a three-year period. Atmos. Environ..

[50] Wang B., Li Y., Tang Z., Cai N., Zhang N., Liu J. (2021). The heavy metals in indoor and outdoor PM_2.5_ from coal-fired and non-coal-fired area. Urban Clim..

[51] Hu Y., You M., Liu G., Dong Z. (2021). Characteristics and potential ecological risks of heavy metal pollution in surface soil around coal-fired power plant. Environ. Earth Sci..

[52] Mor S., Vig N., Ravindra K. (2022). Distribution of heavy metals in surface soil near a coal power production unit: Potential risk to ecology and human health. Environ. Monit. Assess..

[53] Chen X., Balasubramanian R., Zhu Q., Behera S.N., Bo D., Huang X., Xie H., Cheng J. (2016). Characteristics of atmospheric particulate mercury in size-fractionated particles during haze days in Shanghai. Atmos. Environ..

[54] Zhang Y., Liu R., Wang Y., Cui X., Qi J. (2015). Change characteristic of atmospheric particulate mercury during dust weather of spring in Qingdao, China. Atmos. Environ..

[55] Li B., Zhou Z., Xue Z., Wei P., Ren Y., Cao L., Feng X., Yao Q., Ma J., Xu P. (2020). Study on the Pollution Characteristics and Sources of Ozone in Typical Loess Plateau City. Atmosphere.

[56] Liu K., Wu Q., Wang S., Chang X., Tang Y., Wang L., Liu T., Zhang L., Zhao Y., Wang Q.G. (2022). Improved atmospheric mercury simulation using updated gas-particle partition and organic aerosol concentrations. J. Environ. Sci..

[57] Ariya P.A., Amyot M., Dastoor A., Deeds D., Feinberg A., Kos G., Poulain A., Ryjkov A., Semeniuk K., Subir M. (2015). Mercury Physicochemical and Biogeochemical Transformation in the Atmosphere and at Atmospheric Interfaces: A Review and Future Directions. Chem. Rev..

[58] Yawei S., Jianhai L., Junxiu Z., Xiaobo P., Zewu Q. (2021). Epidemiology, clinical presentation, treatment, and follow-up of chronic mercury poisoning in China: A retrospective analysis. BMC Pharmacol. Toxicol..

