# Supplementary material for: Long-Term Variation Characteristics and Health Risks of Atmospheric Hg in the Largest City in Northwestern China
Source: Toxics. 2024 Dec 23;12(12):935. doi: 10.3390/toxics12120935 (PMC11728521; doi:10.3390/toxics12120935)
Supplement: Supplementary file 1 [file toxics-12-00935-s001.zip › toxics-3362558-supplementary.pdf]

Supporting Information

Long-Term Variation Characteristics and Health Risks of Atmospheric Hg in the Largest City in Northwestern China

Yuqi Pang [1], Hongmei Xu [1,*], Mengyun Yang [1], Bin Zhang [1], Liyan Liu [1], Sulin Chen [1], Jing Xue [2], Hui Zhang [3] and Zhenxing Shen [1]

[1] Department of Environmental Science and Engineering, Xi'an Jiaotong University, Xi'an 710049, China
[2] Key Laboratory for Space Bioscience and Biotechnology, School of Life Sciences, Northwestern Polytechnical University, Xi'an 710072, China
[3] State Key Laboratory of Environmental Geochemistry, Institute of Geochemistry, Chinese Academy of Sciences, Guiyang 550081, China
* Correspondence: xuhongmei@xjtu.edu.cn

Table S1. Distribution percentiles of physiological daily inhalation rates (m^3/day) for free-living normal-weight males and females aged 1 to 96 years

Age Group (years)	N	Body Weight [a] (kg)	Physiological Daily Inhalation Rates [b] (m^3/day) Percentile [c]								
		Mean ± SD	Mean ± SD	5th	10th	25th	50th	75th	90th	95th	99th
**Males**											
l to<2	35	10.6±1.1	5.12±0.88	3.68	3.99	4.53	5.12	5.71	6.25	6.56	7.16
2 to<5	25	15.3±3.4	7.60±1.28	5.49	5.95	6.73	7.60	8.47	9.25	9.71	10.59
5 to<7	96	19.8±2.1	8.64±1.23	6.61	7.06	7.81	8.64	9.47	10.21	10.66	11.50
7 to<11	38	28.9±5.6	10.59±1.99	7.32	8.04	9.25	10.59	11.94	13.14	13.87	15.22
11 to<23	30	58.6±13.9	17.23±3.67	11.19	12.53	14.75	17.23	19.70	21.93	23.26	25.76
23 to<30	34	70.9±6.5	17.48±2.81	12.86	13.88	15.59	17.48	19.38	21.08	22.11	24.02
30 to<40	41	71.5±6.8	16.88±2.50	12.77	13.68	15.20	16.88	18.57	20.09	21.00	22.70
40 to<65	33	71.1±7.2	16.24±2.67	11.84	12.81	14.44	16.24	18.04	19.67	20.64	22.46
65 to≤96	50	68.9±6.7	12.96±2.48	8.89	9.79	11.29	12.96	14.63	16.13	17.03	18.72
**Females**											
I to<2	66	10.6±1.3	4.78±0.96	3.20	3.55	4.13	4.78	5.43	6.01	6.36	7.02
2 to<5	36	14.4±3.0	7.06±1.16	5.15	5.57	6.28	7.06	7.84	8.54	8.97	9.76
5 to<7	102	19.7±2.3	8.22±1.31	6.06	6.54	7.34	8.22	9.11	9.90	10.38	11.27
7 to<11	161	28.3±4.4	9.84±1.69	7.07	7.68	8.70	9.84	10.98	12.00	12.61	13.76
11 to<23	87	50.0±8.9	13.28±2.60	9.00	9.94	11.52	13.28	15.03	16.61	17.56	19.33
23 to<30	68	59.2±6.6	13.67±2.28	9.91	10.74	12.13	13.67	15.21	16.59	17.42	18.98
30 to<40	59	58.7±5.9	13.68±1.76	10.78	11.42	12.49	13.68	14.87	15.94	16.58	17.78
40 to<65	58	58.8±5.1	12.31±2.07	8.91	9.66	10.92	12.31	13.70	14.96	15.71	17.12
65 to≤96	45	57.2±7.3	9.80±2.17	6.24	7.02	8.34	9.80	11.27	12.58	13.37	14.85
[a] Measured body weight Normal-weight individuals defined according to the BMI cut-offs.											

[b] Physiological daily inhalation rates were calculated using the following equation: (TDEE + ECG) × H × (V_E/VO_2) × 10^{-3}, where H=0.21L of O_2/Kcal, V_E/VO_2= 27 (Layton, 1993) and ECG = stored daily enegy cost for growth (kcal/day).

[c] Percentiles basedonanormal distribution assumption for age groups.

N =Number of individuals.

SD =Standard deviation.

Source: EPA's Exposure Factors Handbook (EFH), Chapter 6-Inhalation Rates, Table 6-4, https://www.epa.gov/expobox/about-exposure-factors-handbook.

Table S2. Correlation between GEM, GOM and carbonaceous aerosols, elements in $PM_{2.5}$. ** indicates significant correlation at the 0.01 level (double tailed); * indicates significant correlation at the 0.05 level (double tailed)

	**TC**	**OC**	**EC**	**OC1**	**OC2**	**OC3**	**OC4**
**GEM**	0.063	0.035	.110**	-0.034	.099*	0.042	0.049
**GOM**	-0.053	-0.068	-0.015	0.004	-0.064	0.064	0.017
	**EC1**	**EC2**	**OP2**	**OM**	**Na**	**Mg**	**Al**
**GEM**	0.08	-.108*	-0.021	0.035	.230**	.201**	.132**
**GOM**	-.140**	.268**	-.236**	-0.068	-.156**	.140**	.284**
	**Si**	**P**	**S**	**Cl**	**K**	**Ca**	**Sc**
**GEM**	.112**	.181**	.242**	0.022	.128**	0.05	.119*
**GOM**	.309**	.287**	-.304**	-.246**	0.029	.427**	.380**
	**Ti**	**V**	**Cr**	**Mn**	**Fe**	**Ni**	**Cu**
**GEM**	.122**	.121*	.243**	.164**	.145**	.131*	.156**
**GOM**	.325**	.151**	.125**	.140**	.337**	0.053	.121**
	**Zn**	**As**	**Se**	**Br**	**Sr**	**Ba**	**Pb**
**GEM**	.136**	.436**	.244**	.129**	0.079	0.115	.191**
**GOM**	0.042	-0.045	-.180**	-.245**	.233**	.172**	-.192**

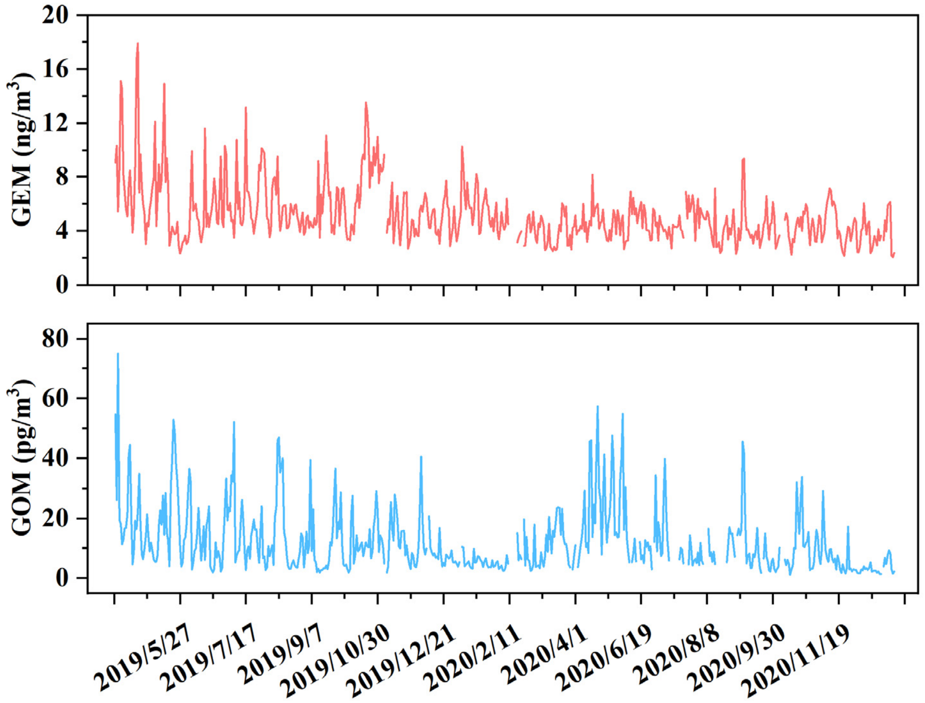

Figure S1. The daily variation of GEM and GOM concentrations from April 2019 to December 2020 in Xi'an

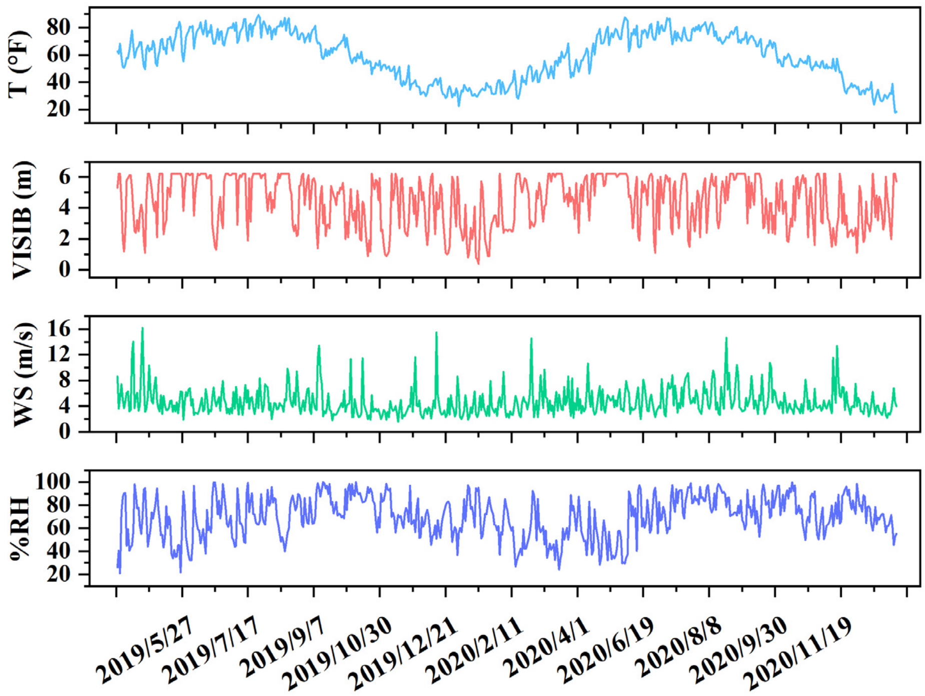

Figure S2. The daily variation of meteorological factors from April 2019 to December 2020 in Xi'an

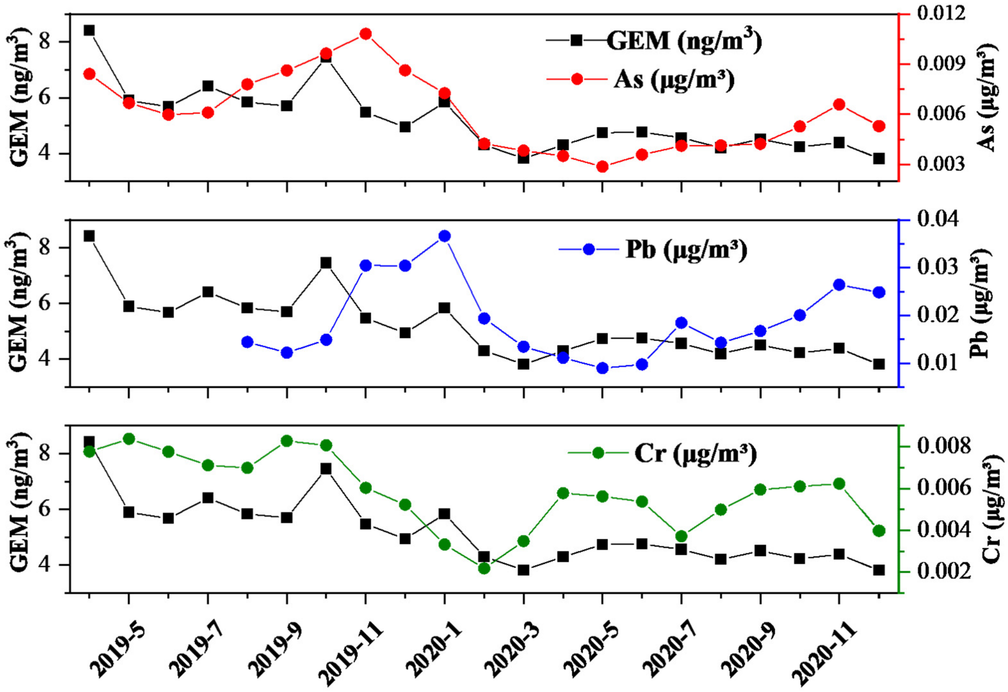

Figure S3. The average monthly variation of heavy metals (As, Pb, Cr) concentrations and GEM during the sampling period
